# Methylphosphonate Oxidation in *Prochlorococcus* Strain MIT9301 Supports Phosphate Acquisition, Formate Excretion, and Carbon Assimilation into Purines

**DOI:** 10.1128/AEM.00289-19

**Published:** 2019-06-17

**Authors:** Oscar A. Sosa, John R. Casey, David M. Karl

**Affiliations:** a Daniel K. Inouye Center for Microbial Oceanography: Research and Education, University of Hawai‘i at Mānoa, Honolulu, Hawaii, USA; Chinese Academy of Sciences

**Keywords:** *Prochlorococcus*, dissolved organic matter, formate, methylphosphonate, oligotrophic ocean, phosphate scarcity, stress adaptation

## Abstract

The marine unicellular cyanobacterium *Prochlorococcus* is an abundant primary producer and widespread inhabitant of the photic layer in tropical and subtropical marine ecosystems, where the inorganic nutrients required for growth are limiting. In this study, we demonstrate that *Prochlorococcus* high-light strain MIT9301, an isolate from the phosphate-depleted subtropical North Atlantic Ocean, can oxidize methylphosphonate (MPn) and hydroxymethylphosphonate (HMPn), two phosphonate compounds present in marine dissolved organic matter, to obtain phosphorus. The oxidation of these phosphonates releases the methyl group as formate, which is both excreted and assimilated into purines in RNA and DNA. Genes encoding the predicted phosphonate oxidative pathway of MIT9301 were predominantly present in *Prochlorococcus* genomes from parts of the North Atlantic Ocean where phosphate availability is typically low, suggesting that phosphonate oxidation is an ecosystem-specific adaptation of some *Prochlorococcus* populations to cope with phosphate scarcity.

**IMPORTANCE** Until recently, MPn was only known to be degraded in the environment by the bacterial carbon-phosphorus (CP) lyase pathway, a reaction that releases the greenhouse gas methane. The identification of a formate-yielding MPn oxidative pathway in the marine planctomycete Gimesia maris (S. R. Gama, M. Vogt, T. Kalina, K. Hupp, et al., ACS Chem Biol 14:735–741, 2019, https://doi.org/10.1021/acschembio.9b00024) and the presence of this pathway in *Prochlorococcus* indicate that this compound can follow an alternative fate in the environment while providing a valuable source of P to organisms. In the ocean, where MPn is a major component of dissolved organic matter, the oxidation of MPn to formate by *Prochlorococcus* may direct the flow of this one-carbon compound to carbon dioxide or assimilation into biomass, thus limiting the production of methane.

## Abstract (precis)

Until recently, MPn was only known to be degraded in the environment by the bacterial carbon-phosphorus (CP) lyase pathway, a reaction that releases the greenhouse gas methane. The identification of a formate-yielding MPn oxidative pathway in the marine planctomycete Gimesia maris (S. R. Gama, M. Vogt, T. Kalina, K. Hupp, et al., ACS Chem Biol 14:735–741, 2019, https://doi.org/10.1021/acschembio.9b00024) and the presence of this pathway in *Prochlorococcus* indicate that this compound can follow an alternative fate in the environment while providing a valuable source of P to organisms. In the ocean, where MPn is a major component of dissolved organic matter, the oxidation of MPn to formate by *Prochlorococcus* may direct the flow of this one-carbon compound to carbon dioxide or assimilation into biomass, thus limiting the production of methane.

## INTRODUCTION

The unicellular cyanobacterium *Prochlorococcus* is the most abundant photoautotroph and a major contributor to primary productivity in tropical and subtropical marine ecosystems worldwide (1, 2). In the oligotrophic Sargasso Sea in the subtropical North Atlantic Ocean and in the eastern Mediterranean Sea, inorganic phosphate (PO_4_^3−^; P_i_ here) has been shown to limit both phytoplankton and bacterial productivity (3–5). In these regions, *Prochlorococcus* displays unique adaptations to cope with P_i_ depletion, including the acquisition of genes that encode proteins to transport and hydrolyze organic phosphorus (P) compounds (6–9).

Phosphonates, reduced organophosphorus compounds with a carbon-phosphorus (CP) covalent bond [P(III) oxidation state], constitute a major component of dissolved organic phosphorus (DOP) in marine surface waters (10–12). Phosphonates make up ∼25% of the P stored in the high-molecular-weight (HMW) fraction of dissolved organic matter (DOM) and are enriched in this pool relative to that in particulate organic matter (12). The CP bond of phosphonates is highly resistant to chemical hydrolysis and to high temperatures (13). However, microorganisms have evolved several enzymatic strategies that can cleave the CP bond of phosphonates. One is the CP lyase multiprotein complex, which employs a radical *S*-adenosylmethionine (SAM) mechanism through an iron-sulfur center (14). There is also a group of phosphonate hydrolases, or phosphonatases, whose substrates contain an electron-withdrawing β-carbonyl group that facilitates bond cleavage (15). Most recently, CP bond cleavage systems that employ an oxidative pathway have been identified and characterized (16–18).

The *Prochlorococcus* high-light-adapted strain MIT9301 and low-light-adapted strain MIT9303 harbor two genes, *phnY* and *phnZ*, predicted to encode a phosphonate oxidative pathway (16–18) colocated with an ATP-binding cassette transporter system for phosphite and phosphonates (19) and with the P_i_ response regulator *phoB* (7). Despite the presence of these genes in *Prochlorococcus* isolates, previous studies have failed to demonstrate *Prochlorococcus* utilization of phosphonates as the sole source of P (20). In this study, we present evidence that MIT9301 can utilize two previously unrecognized phosphonates enriched in marine HMW DOM, methylphosphonate (MPn) and hydroxymethylphosphonate (HMPn) (11). Our findings indicate that *Prochlorococcus* strain MIT9301 metabolizes these phosphonates to obtain P and can also excrete and assimilate the methyl group released during MPn and HMPn CP bond cleavage as formate, consistent with the MPn-specific oxidative mechanism identified in the marine planctomycete Gimesia maris (18). Our results support the hypothesis that phosphonates play an important role in the daily P nutrition of microbial communities in marine environments limited by P_i_ and implicate the degradation of MPn and HMPn by *Prochlorococcus* as a potential source of formate for bacteria.

## RESULTS

**FIG 1:**
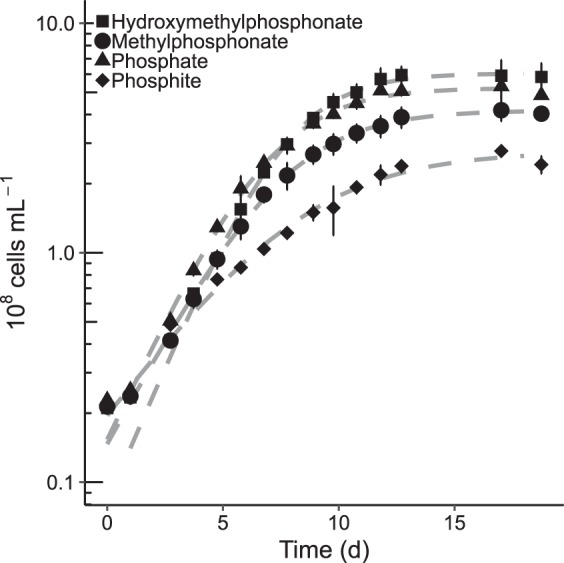
*Prochlorococcus* strain MIT9301 can grow on methylphosphonate or hydroxymethylphosphonate as sole P source. MIT9301 cultures were grown in Pro99 medium containing the indicated P source added at a concentration of 50 μM. Error bars represent one standard deviation of the mean cell density from triplicate cultures. Dashed gray lines represent the modeled logistic growth on each P substrate.

*Prochlorococcus* strain MIT9301 was able to use P_i_, phosphite, MPn, or HMPn as the sole source of P to support its growth (Fig. 1). Two other phosphonate compounds, 2-aminoethylphosphonate (2-AEP) and 2-hydroxyethylphosphonate (2-HEP), did not support the growth of MIT9301 (see Fig. S1 in the supplemental material). In contrast, strain MED4 was unable to grow in medium provided with phosphonates or phosphite and was only able to utilize P_i_ for growth (Fig. S1). The cell yields of MIT9301 cultures in medium containing P_i_ or HMPn were comparable to and greater than those in medium amended with MPn or phosphite as the primary P source, respectively (Fig. 1). The maximum cell yields of P_i_- and HMPn-grown cultures averaged 6 × 10^8^ cells ml^−1^, approximately two times greater than the cell yields of cultures grown on phosphite. The maximum specific growth rates of MIT9301 cultures utilizing P_i_, phosphite, MPn, and HMPn were 0.42 ± 0.03 day^−1^, 0.18 ± 0.01 day^−1^, 0.35 ± 0.01 day^−1^, and 0.39 ± 0.02 day^−1^, respectively.

The rate at which MIT9301 assimilated MPn C into biomass, as determined by [^14^C]MPn labeling experiments, varied with light availability. Assimilation rates were approximately 3 times greater under light conditions than in darkness, 250 ± 58 zmol C cell^−1^ h^−1^ and 89 ± 13 zmol C cell^−1^ h^−1^, respectively. The [^14^C]MPn based assimilation rate served as a proxy for the assimilation rate of P. The assimilation rate of MPn over a full day of growth (4.3 ± 0.2 amol C day^−1^) divided by the cellular P content determined for MIT9301 cultures grown with MPn (20 ± 3 amol P cell^−1^) resulted in a maximum P-specific growth rate of 0.22 day^−1^, which was insufficient to account for the measured growth rate of 0.43 day^−1^ determined for these cultures under the same incubation conditions. Conversion of [^14^C]MPn to ^14^CO_2_ (0.09 ± 0.05 amol C day^−1^) was equal to 2% of the assimilation rate during the 24-h incubation period and accounted for 1.7% of the deficit in observed growth rate.

**FIG 2:**
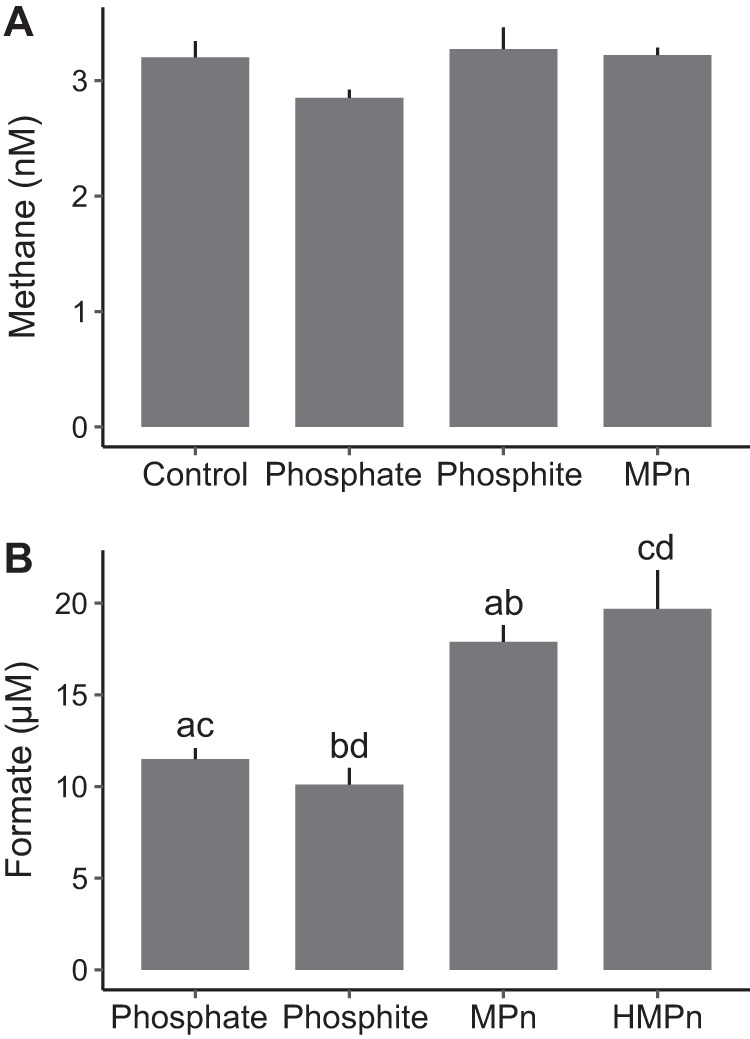
Methane and formate produced by *Prochlorococcus* strain MIT9301 grown with phosphate, phosphite, methylphosphonate (MPn), or hydroxymethylphosphonate (HMPn). (A) Dissolved methane concentrations measured in MIT9301 cultures after 7 days of growth in gas-tight bottles. The control measurement corresponds to the methane concentration at the start of the incubation to account for the solubility of atmospheric methane in the medium. The error bars represent the standard deviations of the means from triplicate cultures. (B) Formate concentrations measured in the supernatant of MIT9301 cultures grown to maximum cell densities for 14 days with the indicated P substrate as sole P source. Formate was not detected in the sterile medium at the start of the incubation. Error bars represent the standard deviations of the means from triplicate cultures. The average formate concentrations in treatments marked with the same lowercase letter (a, b, c, or d) were significantly different (*P* < 0.05).

The methane concentration measured in MIT9301 cultures grown for 7 days with P_i_, phosphite, or MPn did not differ significantly (*P* > 0.05) from the initial methane concentration (the control measurement accounts for the solubility of atmospheric methane in the medium), indicating that methane production was not derived from or induced by these substrates (Fig. 2A). In turn, formate was produced by MIT9301 cultures grown on all P substrates tested (Fig. 2B). Formate was not detected in the sterile medium. The difference in formate concentrations between cultures grown with MPn or HMPn and cultures grown with P_i_ or phosphite was significant (*P* < 2.6 × 10^−4^). The amounts of formate per cell produced in cultures grown with MPn or HMPn (48 ± 0.2 amol cell^−1^) were not significantly different from one another and were 178% greater than in cultures grown with P_i_ or phosphite (27 ± 0.8 amol cell^−1^). The excess formate excreted by cells grown with MPn as the sole P source (21 ± 2 amol cell^−1^) was nearly equal to the P content of these cells (20 ± 3 amol P cell^−1^). Dividing the [^14^C]MPn assimilation rate by the excess formate accounted for 100% (0.21 day^−1^) of the deficit (0.21 day^−1^) between the observed growth rate and the P-specific growth rate. Based on these results, *Prochlorococcus* excreted approximately half (∼49%) of the formate derived from MPn, assimilated most of the remaining into biomass (∼51%), and oxidized a small portion to CO_2_ (<1%).

**FIG 3:**
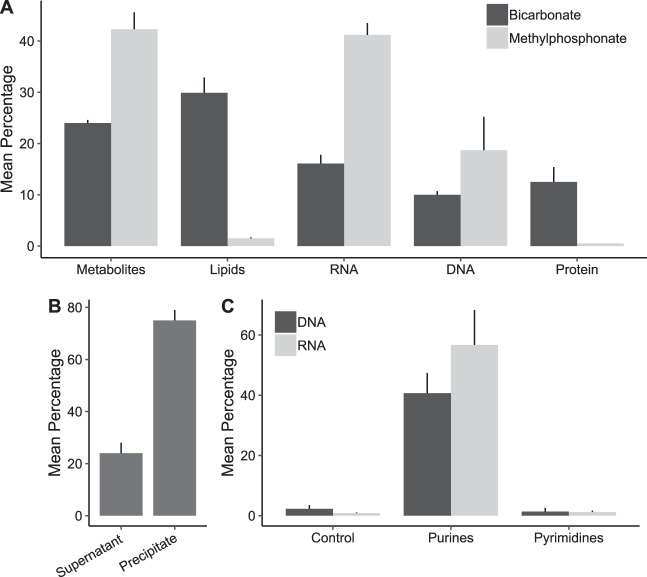
^14^C labeling of *Prochlorococcus* strain MIT9301 biomass with methylphosphonate or bicarbonate tracers. (A) The mean [^14^C]methylphosphonate or [^14^C]bicarbonate radioactivity that was incorporated into each class of macromolecule or extractable fraction, expressed as a percentage of the total radioactivity in intact cell pellets. Error bars represent one standard deviation of the mean from triplicate cell pellets. The metabolite fraction corresponds to cell pellet acid-soluble materials. Fractions are shown in the order in which they were obtained from cell pellets. (B) The mean [^14^C]methylphosphonate radioactivity in acid-soluble metabolites that coprecipitated with Mg(OH)_2_ or that remained in the supernatant. Mean radioactivity is expressed as a percentage of total radioactivity in the acid-soluble fraction. (C) Mean [^14^C]methylphosphonate radioactivity in purines and pyrimidines in DNA or RNA, expressed as a percentage of total DNA or RNA radioactivity. Error bars represent one standard deviation of the mean from triplicate cultures. Controls correspond to the TLC sheet area where samples were spotted.

In exponentially growing cultures of MIT9301, approximately 60% of [^14^C]MPn radiolabel detected in biomass was present in nucleic acids, 41% ± 2% in RNA and 19% ± 6% in DNA (Fig. 3A). Lipids and proteins accounted for 1.5 ± 0.2% and 0.5% of the radiolabel present in biomass, respectively (Fig. 3A). The remaining [^14^C]MPn detected in cells (42% ± 3%) was present as acid-soluble materials (Fig. 3A). In these acid-soluble materials, 75% ± 4% of the radiolabel coprecipitated with authigenic Mg(OH)_2_ (Fig. 3B). P_i_ and ribo- and deoxyribonucleotides are all quantitatively precipitated with authigenic Mg(OH)_2_ (21). In turn, [^14^C]bicarbonate radiolabel was incorporated into all biomass components (24% ± 0.6% in acid-soluble materials, 30% ± 3% in lipids, 16% ± 1.7% in RNA, 10% ± 0.7% in DNA, and 12.5% ± 2.9% in protein) (Fig. 3A).

The alkaline hydrolysis products of RNA (AMP, GMP, CMP, and UMP) isolated from MIT9301 cells grown with [^14^C]MPn were separated by thin-layer chromatography (TLC) to further elucidate the metabolic route of MPn. Radioactivity was detected only in the purine ribonucleotides AMP and GMP (Fig. 3C). The digestion products of DNA isolated from MIT9301 cultures grown with [^14^C]MPn were also separated by TLC. The radiolabel was detected only in the purine nucleosides deoxyadenosine and deoxyguanosine (Fig. 3C).

**FIG 4:**
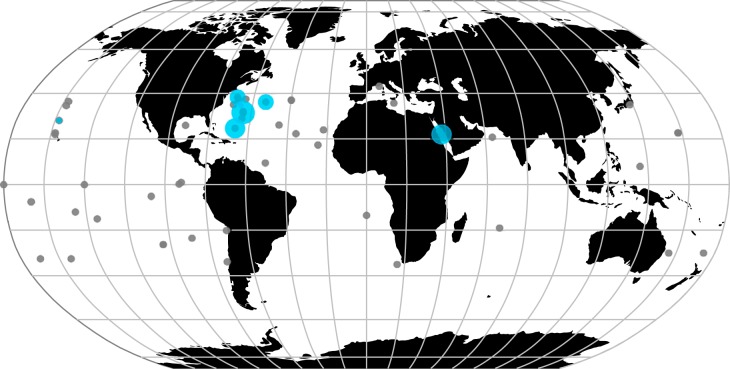
Oceanic distribution of *Prochlorococcus* genomes containing the PhnY-PhnZ phosphonate oxidative pathway. Blue circles indicate sampling locations where genomes had significant matches to PhnY and PhnZ. The circle diameter is proportional to the number of genomes with significant matches in each site. The scale ranges from 1 to 4 genomes. Gray circles indicate locations where the phosphonate oxidative pathway was absent in genomes. The circle diameter scale was not applied to these sites.

Of the 588 *Prochlorococcus* genomes analyzed (excluding MIT9301 and MIT9303), 16 had matches to PhnY and PhnZ of MIT9301 (Table 1). Of these genomes, 15 had matches with >97% protein sequence identity to PhnY and PhnZ of MIT9301 and all belonged to high-light-adapted clades, except for 3 genomes which did not have a clade assignment but were isolated from a depth of 10 m. One additional low-light-adapted genome had matches that shared 99.6% and 100% protein sequence identity with PhnY and PhnZ of MIT9303, respectively. Between MIT9301 and MIT9303, the protein sequences for PhnY and PhnZ shared 48.0% and 49.4% identity, respectively. The geographic locations of *Prochlorococcus* genomes analyzed and of those possessing significant matches to PhnY and PhnZ are shown in Fig. 4. The majority of genomes with PhnY and PhnZ matches were obtained from the North Atlantic Ocean (12 of 16 genomes). Three genomes were obtained from the Red Sea and one from the North Pacific Ocean (Fig. 4). The proportions of *Prochlorococcus* genomes with PhnY and PhnZ were significantly greater in the North Atlantic Ocean than in the North Pacific Ocean (*χ*^2^ = 8.15, *df* = 1; *P* = 0.002151), at 6.4% (of 219 genomes) and 0.5% (of 187 genomes), respectively.

**TABLE 1:** *Prochlorococcus* genomes possessing significant matches to PhnY and PhnZ

Isolate or single cell	IMG ID^*a*^	Clade^*b*^	Depth (m)	Latitude	Longitude	Ocean^*c*^
*Prochlorococcus* strain MIT9301	640069322	HLII	90	34.75	−66.18	NAO
*Prochlorococcus* strain MIT9302	2606217691	HLII	100	34.75	−66.19	NAO
*Prochlorococcus* strain MIT9303	640069323	LLIV	100	34.75	−66.18	NAO
*Prochlorococcus* sp. AG-355-M18	2667527302	HLII	10	31.67	−64.17	NAO
*Prochlorococcus* sp. AG-355-N22	2667527306	HLII	10	31.67	−64.17	NAO
*Prochlorococcus* sp. AG-355-P11	2667527349	HLII	10	31.67	−64.17	NAO
*Prochlorococcus* sp. AG-363-B18	2667527363	HLVI	100	31.67	−64.17	NAO
*Prochlorococcus* sp. AG-388-E21	2716884367	HLI	8	36.20	−53.31	NAO
*Prochlorococcus* sp. AG-388-I18	2716884369	HLI	8	36.20	−53.31	NAO
*Prochlorococcus* sp. AG-412-C21	2716884755	HLII	119	24.71	−67.07	NAO
*Prochlorococcus* sp. AG-412-F02	2716884756	HLVI	119	24.71	−67.07	NAO
*Prochlorococcus* sp. AG-412-L10	2716884761	LLIV	119	24.71	−67.07	NAO
*Prochlorococcus* sp. AG-424-J22	2716884773	HLII	90.8	38.32	−68.87	NAO
*Prochlorococcus* sp. AG-424-P23	2716884420	HLII	90.8	38.32	−68.87	NAO
*Prochlorococcus* sp. AG-670-M15	2716884478	HLII	5	28.14	−158.00	NPO
*Prochlorococcus* sp. RS01	2765235964		10	22.05	37.93	RS
*Prochlorococcus* sp. RS04	2765235965		10	22.05	37.93	RS
*Prochlorococcus* sp. RS50	2765235963		10	22.05	37.93	RS

a IMG ID, Integrated Microbial Genomes genome identification.

b Clades are abbreviated HL for high-light-adapted and LL for low-light-adapted, followed by a Roman numeral.

c Ocean abbreviations: NAO, North Atlantic Ocean; NPO, North Pacific Ocean; and RS, Red Sea.

## DISCUSSION

In this study, we found that *Prochlorococcus* strain MIT9301 can grow on MPn or HMPn as the sole source of P. Our data support the hypothesis that this capability is mediated by the putative phosphonate oxidative pathway encoded by the *phnY* and *phnZ* genes (16–18). Strain MED4, a high-light-adapted strain isolated from the oligotrophic and P_i_-depleted surface waters of the Mediterranean Sea (22), lacks these genes and is accordingly unable to utilize 2-AEP (20). Our results confirmed that MED4 is also unable to grow on MPn as the sole source of P.

**FIG 5:**
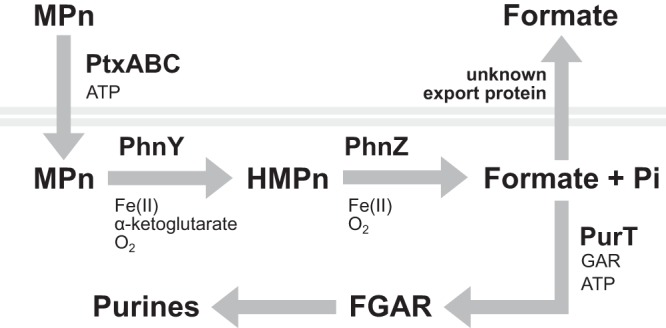
Hypothesized metabolic pathway of methylphosphonate in *Prochlorococcus* strain MIT9301. Methylphosphonate (MPn) is transported across the cell membrane (gray lines) by the PtxABC ATP-binding cassette transport system. MPn carbon is hydroxylated by PhnY, an Fe(II)/α-ketoglutarate-dependent dioxygenase, forming hydroxymethylphosphonate (HMPn). PhnZ, an Fe(II)-dependent oxygenase of the histidine-aspartate hydrolase superfamily, completes the oxidation of MPn to formate and inorganic phosphate (P_i_). Formate can be excreted through the cell membrane by simple diffusion or by an unknown transporter. Formate is also assimilated by PurT to form FGAR (formyl-glycinamide ribose). FGAR is further modified in the synthesis of purine ribonucleotides.

Colocated with *phnY* and *phnZ* in the MIT9301 genome is a four-gene cluster predicted to encode a phosphite ATP-binding cassette transport system (*ptxABC*) and an NAD-dependent phosphite dehydrogenase (*ptxD*) (20). However, *in vitro* studies showed that the periplasmic protein PtxB of this transport system in MIT9301 has greater affinity to MPn than to phosphite, suggesting that it may also function to transport MPn (19). These observations indicate that MIT9301 has the ability to transport and metabolize phosphonates (Fig. 5).

The reaction mediated by PhnY and PhnZ was first characterized by expressing homologs of *phnY* and *phnZ* identified in a marine metagenome in Escherichia coli and performing *in vitro* experiments with the purified proteins and 2-AEP as the substrate (17). The study confirmed that PhnY is an α-ketoglutarate/Fe(II)-dependent dioxygenase and PhnZ is an Fe(II)-dependent enzyme of the histidine-aspartate motif hydrolase superfamily. Through this reaction, PhnY hydroxylates the α-carbon of 2-AEP, which makes the CP bond susceptible to cleavage. PhnZ then completes CP bond oxidation to yield glycine and P_i_ (17). MPn and ethylphosphonate were also tested as the substrates, but these were not oxidized, suggesting that the PhnY and PhnZ pathway can exhibit substrate specificity (17). The *Prochlorococcus* MIT9301 *phnY* and *phnZ* genes were also cloned in E. coli, but these did not confer the ability to grow on phosphonates, including MPn, possibly because the *phnY* and *phnZ* gene sequences were not optimized for expression in the host (20). In this study, we found that MIT9301 was unable to utilize 2-AEP and 2-HEP, indicating that its phosphonate oxidative system may be specific to MPn and HMPn.

Most recently, *phnY* and *phnZ* homologs belonging to the marine planctomycete *G. maris* DSM8797 were expressed in E. coli and shown to confer the ability to specifically oxidize MPn to HMPn and subsequently to formate and P_i_ (18). Based on sequence clustering, the *phnY* and *phnZ* genes in *G. maris* were predicted to be closely related to the *Prochlorococcus phnY* and *phnZ* genes (18). Our finding that MIT9301 can grow on either MPn or HMPn as the sole source of P is consistent with this metabolic pathway. Similar to the phosphonate oxidative mechanism in *G. maris* (18), the oxidation of MPn in *Prochlorococcus* may proceed through an HMPn intermediate, where PhnY catalyzes the hydroxylation of MPn C to form HMPn and PhnZ completes the oxidative cleavage of the CP bond in HMPn to yield P_i_ and formate (Fig. 5). The similar growth rates achieved by MIT9301 in medium containing either P_i_ or phosphonates suggest that phosphonates were rapidly converted to P_i_, consistent with the PhnY-PhnZ oxidative pathway. In contrast, phosphite supported lower growth rates than P_i_ or phosphonates, consistent with previous findings (20).

Formate excretion by *Prochlorococcus* isolates has been reported previously (23). The concentrations of formate we measured in MIT9301 cultures grown on P_i_ or phosphite were similar to those reported for strain MED4 during growth under P_i_-replete conditions (23). However, the concentration of formate in MIT9301 cultures grown on MPn or HMPn was higher than in cultures grown with P_i_ or phosphite, suggesting that the excess formate was derived from these phosphonates. The discrepancy between growth rate estimates based on [^14^C]MPn assimilation and cellular P content compared to the measured growth rate of MIT9301 grown with MPn is also consistent with the conversion of MPn to formate by the PhnY-PhnZ oxidative pathway. When taking into account the excess formate excreted by MIT9301 cultures grown with MPn or HMPn, the adjusted [^14^C]MPn assimilation rate more closely approximated the measured growth rate for these cultures during the radiotracer experiment.

The selective incorporation of MPn C into RNA and DNA, specifically into purines, provides further evidence that MIT9301 oxidizes MPn to formate. The formate produced after MPn and HMPn oxidation can be assimilated into purine nucleotides through the enzyme formyl-glycinamide ribonucleotide (FGAR) synthetase (MIT9301 protein accession ABO17716) encoded by *purT* (Fig. 5). In E. coli, FGAR synthetase directly binds and activates formate by phosphorylation with ATP to form FGAR (24). The tetrahydrofolate (H_4_F) pathway can also provide formyl groups to the purine biosynthetic pathway through the *purN*-encoded phosphoribosylglycinamide formyltransferase (MIT9301 protein accession ABO17591) and the *purH*-encoded aminoimidazole-4-carboxamide ribonucleotide transformylase (MIT9301 protein accession ABO16912), which use formyl-H_4_F as a cofactor. The enzyme formate H_4_F ligase found in some bacteria can produce formyl-H_4_F directly (25). However, we did not identify a gene predicted to encode this function in the genome of MIT9301. Thus, assimilation of [^14^C]MPn-derived formate through FGAR synthetase is most likely responsible for the radiolabeling of RNA and DNA purines and for the observed lack of radiolabeling of other biomolecules.

Gene expression studies of strain MIT9301 have shown that *phnY* and *phnZ* are upregulated upon P_i_ depletion (8), suggesting that P_i_ concentration is an important environmental control on the activity of this biochemical pathway. A previous study found that *Prochlorococcus phnY* and *phnZ* genes were enriched and highly expressed in the lower euphotic zone of the Sargasso Sea in the North Atlantic Ocean and were absent from other ocean regions, such as the eastern Pacific Ocean and the Indian Ocean (20). Our analysis of *Prochlorococcus* genomes confirmed that the *phnY*-*phnZ* phosphonate oxidation pathway is more abundant in populations inhabiting surface waters of the subtropical North Atlantic Ocean than in other regions sampled. This result is consistent with the P_i_ deficiency that distinguishes subtropical North Atlantic Ocean surface waters from other oligotrophic ocean regions (26–29) and with the enrichment of other P metabolism genes in *Prochlorococcus* populations in P_i_-limited environments (6–8) and suggests that phosphonates are an important source of P in these regions (16, 20). Phosphonates appear to be ubiquitous in the ocean and are predominantly present in HMW DOM (12). Thus, *Prochlorococcus* may be have evolved to utilize the MPn and HMPn polysaccharide esters present in HMW DOM, which have been shown to be available to bacteria possessing CP lyase (11, 30). These observations indicate that the *phnY*-*phnZ* phosphonate oxidative pathway may confer to *Prochlorococcus* a competitive advantage in P acquisition, particularly in environments where P_i_ is scarce.

The ability of *Prochlorococcus* to utilize MPn and HMPn may also bring about important interactions with other microorganisms that can affect the flow of C in marine surface waters. For example, numerically abundant groups such as the *Pelagibacterales* clade of the *Alphaproteobacteria* may benefit from the release of formate, since this can be oxidized to CO_2_ to obtain energy (31). Members of this clade can also utilize phosphonates through the CP lyase pathway (32) and may compete with *Prochlorococcus* for this valuable source of P in P_i_-depleted marine surface waters. Because MPn yields methane by the CP lyase pathway, if these bacterial interactions indeed take place in the environment, they have the potential to determine how much MPn is converted to methane rather than to formate, biomass, or CO_2_ in the upper ocean.

In summary, our findings implicate *Prochlorococcus* in the cycling of marine phosphonates through the phosphonate oxidative pathway encoded by *phnY* and *phnZ*. The conversion of MPn and HMPn to formate suggests that this pathway can also impact the flow of C in the environment. *Prochlorococcus* assimilated some of the formate released during phosphonate oxidation into nucleic acids and excreted the rest. The latter process may thus benefit bacteria capable of utilizing formate either as a source of energy or as biomass. The *phnY*-*phnZ* phosphonate oxidative pathway appears to be enriched in *Prochlorococcus* populations in subtropical North Atlantic Ocean surface waters, an environment where P_i_ is scarce. These results provide evidence of the importance of phosphonates as a source of P to bacterial communities in P_i_-depleted marine waters. The contribution of *Prochlorococcus* and other bacterial groups possessing this pathway to the turnover of MPn and HMPn in the ocean has yet to be determined.

## MATERIALS AND METHODS

### Culture conditions.

Axenic *Prochlorococcus* cultures of strains MIT9301 and MED4 were obtained from the Chisholm Laboratory at MIT and the National Center for Marine Algae and Microbiota (Maine, USA), respectively. Cultures were grown in Pro99 medium (33) prepared with open ocean surface seawater collected at the Hawaii Ocean Time-series study site station ALOHA (22°45′N, 158°W). Seawater samples were filtered through a 0.22-μm-pore-size membrane and autoclaved. Nutrient and trace metal amendments for Pro99 medium (33) were prepared in autoclaved ultrapure water, filtered through a 0.1-μm-pore-size membrane, and stored frozen. Unless indicated otherwise, cultures were incubated at 24°C under an alternating 12-h light-dark cycle with an irradiance of 30 μmol photons m^−2^ s^−1^ during the light period. All data reported correspond to the means and standard deviations from biological triplicates. The compounds tested to support growth of *Prochlorococcus* cultures as the sole source of P included phosphite (>98%, 04283; Sigma-Aldrich), MPn (98%, A12619; Alfa Aesar), HMPn (95%, sc-477559; Santa Cruz Biotechnologies), 2-AEP (99%, 268674; Sigma-Aldrich), and 2-HEP (>95%; BOC Sciences).

### Culture purity tests.

*Prochlorococcus* cultures were tested for microbial contamination by inoculating samples in ProAC (34), MPTB (35), and ProMM (36) purity broths, as well as in lysogeny broth and marine broth (yeast extract and peptone, Difco 2216; Becton, Dickinson). Purity media were inoculated with 0.5 ml of culture, incubated at 25°C on a shaker incubator (200 rpm) for 1 to 3 weeks, and inspected for contaminants by measuring optical density at a wavelength of 600 nm. Cultures were also plated in marine broth agar and inspected directly by flow cytometry to compare total cells detected by autofluorescence and by SYBR green I staining.

### Comparison of growth supported by different P substrates.

We compared the growth rates and cell yields of MIT9301 cultures in Pro99 medium containing P_i_, phosphite, MPn, or HMPn as the primary P source. To inoculate media, *Prochlorococcus* cells maintained in Pro99 medium containing P_i_ were harvested by centrifugation (5 min at 10,000 × *g*). The supernatant was discarded and the pellet washed twice with sterile oligotrophic surface seawater before inoculating cells into 5 to 10 ml of medium. Culture growth was monitored by flow cytometry on a Guava easyCyte 8HT system (EMD Millipore) using chlorophyll autofluorescence to detect *Prochlorococcus* cells. Growth rates were calculated based on the slope of the linear interval spanning exponential growth according to reference 37. Logistic growth was modeled with the logit function of the car package for the R statistical software.

### Total cellular phosphorus determination.

Cellular P of *Prochlorococcus* was determined with the particulate P method based on the Murphy-Riley molybdenum blue procedure (38). To collect cells, 5 to 6 ml of culture was filtered through 25-mm-diameter glass fiber filters (Whatman GF75, 0.3-μm nominal pore size) and rinsed with three volumes of sterile seawater. Filters were dried and stored in combusted and acid-cleaned borosilicate glass tubes at −20°C. Filters were combusted at 450°C for 4.5 h. P_i_ was extracted by suspending filters in 10 ml of 0.15 N hydrochloric acid for 1 h. Samples were centrifuged at 1,000 × *g*, and 5 ml of supernatant was transferred to combusted acid-cleaned glass tubes for colorimetric determination of P_i_ with 0.5 ml of molybdenum blue reaction mixture. The reaction mixture consisted of an aqueous solution of 5 N sulfuric acid (A300-212; Fisher), 30% ammonium molybdate (A674-S00; Fisher), 5.4% ascorbic acid (A6 I-100; Fisher), and 0.136% potassium antimonyl tartrate (Mallinckrodt 2388) mixed in volumetric proportions of 5:2:2:1. P_i_ standards were prepared in 0.15 N hydrochloric acid and treated with molybdenum blue reaction mixture. Samples were allowed to react for 1 h and were transferred to a 10-cm cell for absorption measurements at 880 nm on a Beckman DU 640 spectrophotometer.

### Methylphosphonate radiotracer experiments.

The radiotracer [^14^C]MPn was custom synthesized by ViTrax (Placentia, CA, USA) with a specific activity of 59 Ci mol^−1^ and a concentration of 16.95 mM. To determine [^14^C]MPn assimilation or conversion to ^14^CO_2_, MIT9301 cultures were pregrown in Pro99 medium containing 50 μM MPn as the sole P source to cell densities of >1 × 10^8^ cells ml^−1^ and then were inoculated into larger volume samples for incubations with radiotracer. For these experiments, [^14^C]MPn was added at 0.1 μCi ml^−1^ (equal to 1.7 μM MPn), and cultures were incubated at 24°C under an alternating 12-h light-dark cycle and an irradiance of 45 μmol photons m^−2^ s^−1^. To determine [^14^C]MPn assimilation into biomass, 30 ml of MIT9301 culture was filtered through 25-mm-diameter glass fiber filters (Whatman GF75) and rinsed subsequently with three volumes of sterile seawater. Filters were recovered, treated with 10 ml of scintillation cocktail (LLC Ultima Gold; Perkin Elmer), and radioassayed by liquid scintillation counting and quench corrected to determine disintegrations per minute (dpm) in each sample. To determine [^14^C]MPn conversion to ^14^CO_2_, 30-ml cultures were incubated in 200-ml glass vials sealed with a rubber septum fitted with a center well containing a fluted piece of filter paper (Whatman no. 2). After the incubation period, the filter paper inside the bottle was treated with 200 μl of 2-phenethylamine (PEA) (>99.5%, 407267; Sigma-Aldrich), and the samples were acidified with 3 ml of 4 N sulfuric acid to release all dissolved carbonate species as CO_2_ as described previously (39). PEA and sulfuric acid were injected through the septum using a syringe and needle. Samples were distilled for 1 day to allow PEA to trap CO_2_ by precipitation as carbonate. The filter paper containing the trapped ^14^CO_2_ was then recovered and radioassayed by liquid scintillation counting as described for ^14^C assimilation measurements. Assimilation and CO_2_ conversion rates were obtained by dividing sample radioactivity by the specific activity of MPn, adjusted by the concentration added as radiotracer, and by dividing by the incubation period. Cell-specific rates were obtained by normalizing to cell density measurements obtained by flow cytometry.

### Radiolabeling and macromolecular fractionation.

Biomass of MIT9301 cultures was partitioned into acid-soluble intracellular metabolites, lipids, RNA, DNA, and protein to assess the fate of MPn or bicarbonate radiotracers in cells following the procedure outlined in reference 40, with several modifications. MIT9301 cultures were grown in Pro99 medium containing 50 μM MPn as the primary P source. During exponential growth, when cultures reached approximately 1 × 10^8^ cells ml^−1^, samples were spiked with 0.25 μCi ml^−1^ of [^14^C]MPn or with 2 μCi ml^−1^ of [^14^C]NaHCO_3_ (MP Biomedicals). Cultures were incubated with radiotracer for 3 days. To harvest cell biomass, triplicate 4-ml culture samples were pelleted by centrifugation (10 min at 10,000 × *g*), the supernatant was carefully discarded, and the pellet was washed once with sterile Pro99 MPn medium. A set of cell pellets was set aside for total radioactivity determinations. For biomass fractionation, cell pellets were resuspended in 1 ml of cold (4°C) 5% trichloroacetic acid (TCA) and treated with 5 μg of nonradioactive carrier DNA (D1626, salmon sperm DNA; Sigma-Aldrich), RNA (10109223001, yeast RNA; Roche), lipids (L5146 lipid mixture; Sigma-Aldrich), and protein (05470, bovine serum albumin; Sigma-Aldrich) to aid with separations. Acid-soluble metabolites were extracted at 4°C for 1 h. Samples were centrifuged at 28,000 × *g* for 10 min to precipitate all acid-insoluble materials. The acid-insoluble materials were washed twice with cold TCA, and the supernatants were pooled for radioassaying by liquid scintillation counting. After TCA extraction, the residual materials were treated with 95% ethanol at room temperature for 20 min. Samples were centrifuged at 28,000 × *g* for 10 min to precipitate ethanol-insoluble materials, and the supernatant was recovered. Samples were washed with 1 ml of ethanol, and supernatants were pooled for radioassaying by liquid scintillation counting. The residual ethanol was evaporated by immersing samples 1 to 2 min in a boiling water bath. To hydrolyze RNA, the dry samples were treated with 0.5 ml of 1 N NaOH and incubated 1 h at 37°C. Samples were neutralized with 0.5 ml of 1 M HCl and 5% TCA. Samples were centrifuged at 28,000 × *g* for 10 min, and the supernatant was recovered. Samples were washed twice with cold TCA, and the supernatants were pooled for radioassaying. Samples were also washed with cold ethanol, and the residual ethanol was removed by evaporation. To hydrolyze DNA, the dry samples were treated with 1 ml of 5% TCA and boiled for 30 min. The supernatants containing hydrolyzed DNA were recovered. Samples were washed twice with TCA, and the supernatants were pooled for radioassaying. Finally, to hydrolyze proteins, dry samples were treated with 1 ml of 1 N NaOH and incubated 18 h at 37°C. Samples were centrifuged and washed twice with 1 N NaOH, and the supernatants were combined for radioassaying. Macromolecular fractions were treated with 10 ml of liquid scintillation cocktail (LLC Ultima Gold; Perkin Elmer). The incorporation of radiolabel into each cellular fraction was expressed as a percentage of the total radiolabel associated with intact cell pellets washed with sterile Pro99 medium containing MPn to remove the radiotracer.

### Separation of cell pellet acid-soluble materials with brucite.

Nucleoside triphosphates and diphosphates were separated from cell pellet acid-soluble extracts by coprecipitation with authigenic brucite [Mg(OH)_2_] (21). To form Mg(OH)_2_, 1 ml of 5% TCA-containing acid-soluble material was treated with 0.1 ml of 1 M MgCl_2_ and 0.4 ml of 1 M NaOH. Samples were centrifuged for 20 min at 1,000 × *g*, the supernatant was removed, and the Mg(OH)_2_ pellet was dissolved in 1 ml of 0.1 M HCl. Supernatants and dissolved Mg(OH)_2_ samples were radioassayed separately in 5 ml of liquid scintillation cocktail.

### Concentration of RNA hydrolysis products.

The RNA hydrolysis procedure (after cold TCA and ethanol extractions) was modified to provide a more concentrated solution of ribonucleotides from ^14^C-labeled MIT9301 cell pellets. Dry cell pellets were treated with 20 μl of 1 N NaOH, instead of 1 ml, and incubated at 37°C for 1 h. Samples were neutralized with 20 μl of 1 M HCl, and TCA was added to a final concentration of 5%. Samples were centrifuged to recover the hydrolyzed-RNA-containing supernatant.

### DNA isolation and enzymatic digestion.

DNA from ^14^C-labeled MIT9301 cultures was isolated by lysing cells in 0.5 ml of sucrose solution containing lysozyme (1 mg ml^−1^) and RNase (0.2 mg ml^−1^) at 37°C for 1 h. The lysate was then treated with proteinase K (0.8 mg ml^−1^) and 1% SDS and heated at 55°C for 2 h. Cell debris was precipitated with 60 μl of sodium acetate solution (3 M, pH 5.2) at −20°C for 30 min and centrifuged at 28,000 × *g* for 10 min at 4°C. The DNA-containing supernatant was treated with 1 ml of 95% ethanol, incubated at −20°C for 1 h, and centrifuged at 4°C. The DNA pellet was washed with 95% ethanol and again with 70% ethanol. Ethanol was allowed to air dry, and the sample was dissolved in 40 μl of water. DNA was further purified using silica spin columns (D4068T, Quick-DNA Miniprep Plus kit; Zymo Research). DNA was eluted in 50 μl of elution buffer supplied by the kit and treated with a nucleoside digestion mixture (M0649S; New England BioLabs) at 37°C for 1 h to digest DNA into deoxynucleosides.

### Thin-layer chromatography of hydrolyzed RNA and DNA.

The resulting ribonucleotides of base-hydrolyzed RNA and deoxynucleosides of enzymatically digested DNA were separated by a two-dimensional thin-layer chromatography (TLC) procedure (41). Before resolution of samples by TLC, 5 to 10 μl of hydrolyzed RNA sample was mixed with an equal volume of a 25 mM solution of AMP, GMP, CMP, and UMP, and digested DNA was concentrated to 5 μl in a speed vacuum and mixed with an equal volume of a 25 mM solution of deoxyadenosine, deoxyguanosine, deoxycytosine, and deoxythyamine. The retention factor of each nucleotide and nucleoside standard was determined separately by TLC. All nucleotide and nucleoside standards were obtained from Sigma-Aldrich. The mixtures were spotted onto a separate polyethyleneimine (PEI) cellulose TLC sheet (10 cm by 10 cm) treated with a UV-fluorescent indicator (EMD Millipore). The first dimension was developed with a solvent consisting of 0.75 M Tris, 0.45 M HCl, and 0.5 M LiCl (pH 8). Sheets were soaked briefly in methanol and allowed to dry. The second dimension was developed with a solvent consisting of saturated ammonium sulfate solution (74 g), ammonium hydrogen sulfate (0.4 g), Na_2_-EDTA (4 g), and 100 ml of ultrapure water (pH 3.5). The location of each nucleotide or nucleoside was detected by exposing the TLC sheets to UV light. Chromatogram spots of approximately 1 cm in diameter were cut out with scissors for radioassaying in 5 ml of liquid scintillation cocktail.

### Detection of methane in cultures.

MIT9301 was grown in Pro99 medium containing 50 μM P_i_, phosphite, or MPn. Cultures were prepared in sterile, acid-cleaned borosilicate bottles with no headspace and crimp-sealed with Teflon-lined septa and aluminum collars. To end incubations, bottles were poisoned with 0.1 ml of a 7% mercuric chloride solution. Dissolved methane in cultures was measured by gas chromatography using a gas stripping and cryo-trap concentration method as described previously (11, 30).

### Detection of formate in cultures.

MIT9301 was grown in Pro99 medium containing 50 μM P_i_, phosphite, MPn, or HMPn to maximum cell densities. Cultures were grown for 14 days at 24°C under a 12-h light-dark cycle with an irradiance of 45 μmol photons m^−2^ s^−1^ during the light period. Formate produced by MIT9301 cultures was measured in 50 μl of supernatant using an enzymatic and colorimetric assay (MAK059; Sigma-Aldrich) in a microwell plate format. Cells were removed from 1-ml culture samples by centrifugation at 10,000 × *g* for 15 min to obtain supernatants. The assay calibration (0 to 10 nmol per well) was performed in a matrix consisting of the same seawater medium used for the cultivation of *Prochlorococcus*. The formate standard was provided in the kit. Sample absorbance was measured at 450 nm on a VICTOR X3 2030 multilabel plate reader (Perkin Elmer).

### Statistical analysis.

Comparisons of the mean methane and formate concentrations measured in cultures and formate concentrations normalized by cell densities were performed with one-way analyses of variance (ANOVAs) and Tukey’s tests, and statistical significance was determined at a *P* value of ≤0.05.

### Genome analysis.

The coding DNA sequences of 588 annotated *Prochlorococcus* isolates or single-cell genomes were downloaded from the Joint Genome Institute’s Integrated Microbial Genomes (IMG) system on 4 January 2019. The majority of single-cell genomes were obtained from the GEOTRACES program data set and additional oceanographic research cruises (42). Genomes unscreened for contamination were not considered. The metadata associated with each genome were used to determine their biogeographic sampling location. BLASTp (v2.6.0+) was used to identify protein sequences similar to PhnY (GenBank accession numbers ABO17878 and ABM77874) and PhnZ (GenBank accession numbers ABO17879 and ABM77875) of *Prochlorococcus* strain MIT9301 (GenBank accession number CP000576.1) and MIT9303 (GenBank accession number CP000554.1) genomes, respectively. Comparison of the proportions of genomes containing PhnY and PhnZ between biogeographic locations was performed with a two-proportion z-test.

## Supplementary Material

Supplemental file 1

## References

[1] Biller SJ, Berube PM, Lindell D, Chisholm SW 2015 *Prochlorococcus*: the structure and function of collective diversity. Nat Rev Microbiol 13:13–27. doi:10.1038/nrmicro3378. 25435307

[2] Partensky F, Hess WR, Vaulot D 1999 *Prochlorococcus*, a marine photosynthetic prokaryote of global significance. Microbiol Mol Biol Rev 63:106–127. 10066832 10.1128/mmbr.63.1.106-127.1999 PMC98958

[3] Cotner JB, Ammerman JW, Peele ER, Bentzen E 1997 Phosphorus-limited bacterioplankton growth in the Sargasso Sea. Aquat Microb Ecol 13:141–149. doi:10.3354/ame013141.

[4] Pinhassi J, Gómez-Consarnau L, Alonso-Sáez L, Sala M, Vidal M, Pedrós-Alió C, Gasol J 2006 Seasonal changes in bacterioplankton nutrient limitation and their effects on bacterial community composition in the NW Mediterranean Sea. Aquat Microb Ecol 44:241–252. doi:10.3354/ame044241.

[5] Thingstad TF, Rassoulzadegan F 1995 Nutrient limitations, microbial food webs, and “biological C-pumps”: suggested interactions in a P-limited Mediterranean. Mar Ecol Prog Ser 117:299–306. doi:10.3354/meps117299.

[6] Martiny AC, Huang Y, Li W 2009 Occurrence of phosphate acquisition genes in *Prochlorococcus* cells from different ocean regions. Environ Microbiol 11:1340–1347. doi:10.1111/j.1462-2920.2009.01860.x. 19187282

[7] Martiny AC, Coleman ML, Chisholm SW 2006 Phosphate acquisition genes in *Prochlorococcus* ecotypes: evidence for genome-wide adaptation. Proc Natl Acad Sci U S A 103:12552–12557. doi:10.1073/pnas.0601301103. 16895994 PMC1567916

[8] Coleman ML, Chisholm SW 2010 Ecosystem-specific selection pressures revealed through comparative population genomics. Proc Natl Acad Sci U S A 107:18634–18639. doi:10.1073/pnas.1009480107. 20937887 PMC2972931

[9] Krumhardt KM, Callnan K, Roache-Johnson K, Swett T, Robinson D, Reistetter EN, Saunders JK, Rocap G, Moore LR 2013 Effects of phosphorus starvation versus limitation on the marine cyanobacterium *Prochlorococcus* MED4 I: uptake physiology. Environ Microbiol 15:2114–2128. doi:10.1111/1462-2920.12079. 23387819

[10] Young CL, Ingall ED 2010 Marine dissolved organic phosphorus composition: insights from samples recovered using combined electrodialysis/reverse osmosis. Aquat Geochem 16:563–574. doi:10.1007/s10498-009-9087-y.

[11] Repeta DJ, Ferrón S, Sosa OA, Johnson CG, Repeta LD, Acker M, DeLong EF, Karl DM 2016 Marine methane paradox explained by bacterial degradation of dissolved organic matter. Nat Geosci 9:884–887. doi:10.1038/ngeo2837.

[12] Kolowith LC, Ingall ED, Benner R 2001 Composition and cycling of marine organic phosphorus. Limnol Oceanogr 46:309–320. doi:10.4319/lo.2001.46.2.0309.

[13] Freedman LD, Doak GO 1957 The preparation and properties of phosphonic acids. Chem Rev 57:479–523. doi:10.1021/cr50015a003.

[14] Kamat SS, Williams HJ, Dangott LJ, Chakrabarti M, Raushel FM 2013 The catalytic mechanism for aerobic formation of methane by bacteria. Nature 497:132–136. doi:10.1038/nature12061. 23615610

[15] Quinn JP, Kulakova AN, Cooley NA, McGrath JW 2007 New ways to break an old bond: the bacterial carbon-phosphorus hydrolases and their role in biogeochemical phosphorus cycling. Environ Microbiol 9:2392–2400. doi:10.1111/j.1462-2920.2007.01397.x. 17803765

[16] Martinez A, Tyson GW, Delong EF 2010 Widespread known and novel phosphonate utilization pathways in marine bacteria revealed by functional screening and metagenomic analyses. Environ Microbiol 12:222–238. doi:10.1111/j.1462-2920.2009.02062.x. 19788654

[17] McSorley FR, Wyatt PB, Martinez A, Delong EF, Hove-Jensen B, Zechel DL 2012 PhnY and PhnZ comprise a new oxidative pathway for enzymatic cleavage of a carbon-phosphorus bond. J Am Chem Soc 134:8364–8367. doi:10.1021/ja302072f. 22564006

[18] Gama SR, Vogt M, Kalina T, Hupp K, Hammerschmidt F, Pallitsch K, Zechel DL 2019 An oxidative pathway for microbial utilization of methylphosphonic acid as a phosphate source. ACS Chem Biol 14:735–741. doi:10.1021/acschembio.9b00024. 30810303

[19] Feingersch R, Philosof A, Mejuch T, Glaser F, Alalouf O, Shoham Y, Béjà O 2012 Potential for phosphite and phosphonate utilization by *Prochlorococcus*. ISME J 6:827–834. doi:10.1038/ismej.2011.149. 22011717 PMC3309357

[20] Martínez A, Osburne MS, Sharma AK, Delong EF, Chisholm SW 2012 Phosphite utilization by the marine picocyanobacterium *Prochlorococcus* MIT9301. Environ Microbiol 14:1363–1377. doi:10.1111/j.1462-2920.2011.02612.x. 22004069

[21] Thompson-Bulldis A, Karl D 1998 Application of a novel method for phosphorus determinations in the ologotrophic North Pacific Ocean. Limnol Oceanogr 43:1565–1577. doi:10.4319/lo.1998.43.7.1565.

[22] Partensky F, Hoepffner N, Li WKW, Ulloa O, Vaulot D 1993 Photoacclimation of *Prochlorococcus* sp. (Prochlorophyta) strains isolated from the North Atlantic and the Mediterranean Sea. Plant Physiol 101:285–296. doi:10.1104/pp.101.1.285. 12231684 PMC158675

[23] Bertilsson S, Berglund O, Pullin MJ, Chisholm SW 2005 Release of dissolved organic matter by *Prochlorococcus*. Vie Milieu Paris 55:225–231.

[24] Marolewski AE, Mattia KM, Warren MS, Benkovic SJ 1997 Formyl phosphate: a proposed intermediate in the reaction catalyzed by *Escherichia coli* PurT GAR transformylase. Biochemistry 36:6709–6716. doi:10.1021/bi962961p. 9184151

[25] Whitehead TR, Park M, Rabinowitz JC 1988 Distribution of 10-formyltetrahydrofolate synthetase in eubacteria. J Bacteriol 170:995–997. doi:10.1128/jb.170.2.995-997.1988. 3257484 PMC210755

[26] Wu J, Sunda WG, Boyle EA, Karl DM 2000 Phosphate depletion in the western North Atlantic Ocean. Science 289:759–762. doi:10.1126/science.289.5480.759. 10926534

[27] Cavendar-Bares KK, Karl DM, Chisholm SW 2001 Nutrient gradients in the western North Atlantic Ocean: Relationship to microbial community structure and comparison to pattern in the Pacific Ocean. Deep Sea Res Part I Oceanogr Res Pap 48:2373–2395. doi:10.1016/S0967-0637(01)00027-9.

[28] Ammerman JW, Hood RR, Case DA, Cotner JB 2003 Phosphorus deficiency in the Atlantic: An emerging paradigm in oceanography. Eos (Washington DC) 84:165–170. doi:10.1029/2003EO180001.

[29] Moore CM, Mills MM, Arrigo KR, Berman-Frank I, Bopp L, Boyd PW, Galbraith ED, Geider RJ, Guieu C, Jaccard SL, Jickells TD, La Roche J, Lenton TM, Mahowald NM, Marañón E, Marinov I, Moore JK, Nakatsuka T, Oschlies A, Saito MA, Thingstad TF, Tsuda A, Ulloa O 2013 Processes and patterns of oceanic nutrient limitation. Nat Geosci 6:701–710. doi:10.1038/ngeo1765.

[30] Sosa OA, Repeta DJ, Ferrón S, Bryant JA, Mende DR, Karl DM, DeLong EF 2017 Isolation and characterization of bacteria that degrade phosphonates in marine dissolved organic matter. Front Microbiol 8:1786. doi:10.3389/fmicb.2017.01786. 29085339 PMC5649143

[31] Sun J, Steindler L, Thrash JC, Halsey KH, Smith DP, Carter AE, Landry ZC, Giovannoni SJ 2011 One carbon metabolism in SAR11 pelagic marine bacteria. PLoS One 6:e23973. doi:10.1371/journal.pone.0023973. 21886845 PMC3160333

[32] Carini P, White AE, Campbell EO, Giovannoni SJ 2014 Methane production by phosphate-starved SAR11 chemoheterotrophic marine bacteria. Nat Commun 5:4346. doi:10.1038/ncomms5346. 25000228

[33] Moore LR, Coe A, Zinser ER, Saito MA, Sullivan MB, Lindell D, Frois-Moniz K, Waterbury J, Chisholm SW 2007 Culturing the marine cyanobacterium *Prochlorococcus*. Limnol Oceanogr Methods 5:353–362. doi:10.4319/lom.2007.5.353.

[34] Morris JJ, Kirkegaard R, Szul MJ, Johnson ZI, Zinser ER 2008 Facilitation of robust growth of *Prochlorococcus* colonies and dilute liquid cultures by “helper” heterotrophic bacteria. Appl Environ Microbiol 74:4530–4534. doi:10.1128/AEM.02479-07. 18502916 PMC2493173

[35] Saito MA, Moffett JW, Chisholm SW, Waterbury JB 2002 Cobalt limitation and uptake in *Prochlorococcus*. Limnol Oceanogr 47:1629–1636. doi:10.4319/lo.2002.47.6.1629.

[36] Berube PM, Biller SJ, Kent AG, Berta-Thompson JW, Roggensack SE, Roache-Johnson KH, Ackerman M, Moore LR, Meisel JD, Sher D, Thompson LR, Campbell L, Martiny AC, Chisholm SW 2015 Physiology and evolution of nitrate acquisition in *Prochlorococcus*. ISME J 9:1195–1207. doi:10.1038/ismej.2014.211. 25350156 PMC4409163

[37] Wood AM, Everroad RC, Wingard LM 2005 Measuring growth rates in microalgal cultures, p 269–285. *In* Andersen R (ed), Algal culturing techniques. Elsevier Academic Press, Boston, MA.

[38] Karl DM, Björkman KM 2001 Phosphorus cycle in seawater: dissolved and particulate pool inventories and selected phosphorus fluxes, p 239–270. *In* Paul JH (ed), Methods in microbiology. Academic Press, Cambridge, MA.

[39] Casey JR, Falkowski PG, Karl DM 2015 Substrate selection for heterotrophic bacterial growth in the sea. Mar Chem 177:349–356. doi:10.1016/j.marchem.2015.06.032.

[40] Karl DM 1982 Selected nucleic acid precursors in studies in aquatic microbial ecology. Appl Environ Microbiol 44:891–902. 16346114 10.1128/aem.44.4.891-902.1982 PMC242114

[41] Bochner BR, Ames BN 1982 Complete analysis of cellular nucleotides by two-dimensional thin layer chromatography. J Biol Chem 257:9759–9769. 6286632

[42] Berube PM, Biller SJ, Hackl T, Hogle SL, Satinsky BM, Becker JW, Braakman R, Collins SB, Kelly L, Berta-Thompson J, Coe A, Bergauer K, Bouman HA, Browning TJ, De Corte D, Hassler C, Hulata Y, Jacquot JE, Maas EW, Reinthaler T, Sintes E, Yokokawa T, Lindell D, Stepanauskas R, Chisholm SW 2018 Data descriptor: single cell genomes of *Prochlorococcus*, *Synechococcus*, and sympatric microbes from diverse marine environments. Sci Data 5:180154. doi:10.1038/sdata.2018.154. 30179231 PMC6122165

