# Supplementary material for: Methylphosphonate Oxidation in Prochlorococcus Strain MIT9301 Supports Phosphate Acquisition, Formate Excretion, and Carbon Assimilation into Purines
Source: Appl Environ Microbiol. 2019 Jun 17;85(13):e00289-19. doi: 10.1128/AEM.00289-19 (PMC6581173; doi:10.1128/AEM.00289-19)
Supplement: Supplemental file 1 [file AEM.00289-19-s0001.pdf]

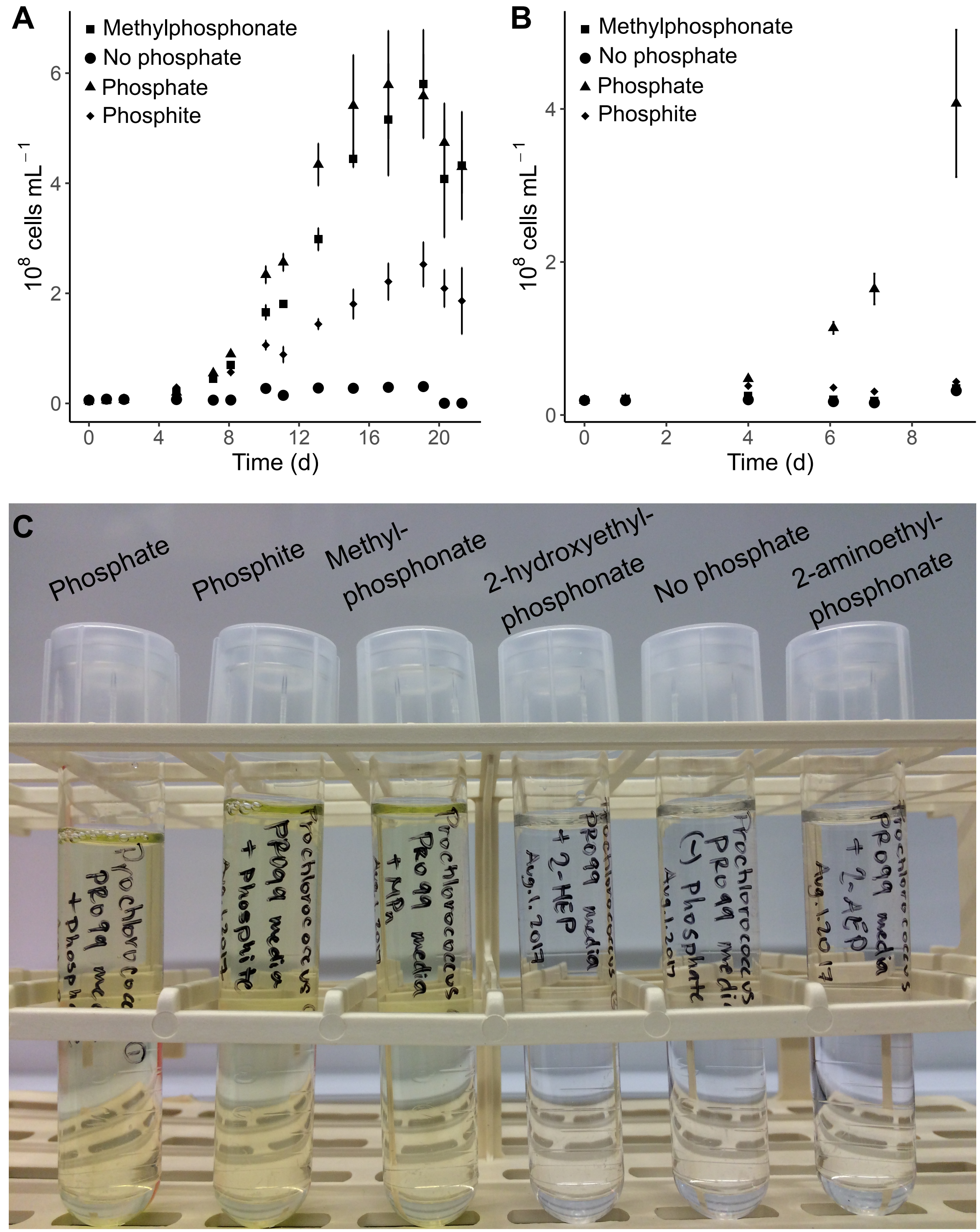

FIG S1. Growth of *Prochlorococcus* strains MIT9301 and MED4.
A. MIT9301 growth on phosphate, phosphite, and methylphosphonate. MIT9301 fails to grow in the absence of phosphate. **B.** MED4 growth on phosphate. MED4 fails to grow on phosphite, methylphosphonate, and in the absence of phosphate. **C.** MIT9301 also fails to grow on 2-hydroxyethylphosphonate and 2-aminoethylphosphonate as indicated by the lack of cell production as in cultures with no phosphate.
